# The economic superorganism in the complexity of evolution

**DOI:** 10.1098/rstb.2021.0417

**Published:** 2023-03-13

**Authors:** Lisi Krall

**Affiliations:** Professor of Economics, SUNY Cortland, Cortland¸ NY 13045, USA

**Keywords:** agriculture, evolutionary transitions, culture, superorganism, economic superorganism, capitalism

## Abstract

The transition to grain agriculture restructured human societies, creating a new whole, an economic superorganism. *Homo sapiens* became expansionary, structurally interdependent in material life, and a duality between them and Earth was created that had not previously existed. Yet *H. sapiens* are not the only species to make the transition to agriculture. Cross-species comparisons create an opening for a movement toward a focus on the universal and powerful agricultural system as a unique expression of the evolution of species cooperation. This shifts the focus around human social evolution away from culture and toward the formation and power of the economic system that took hold with the cultivation of annual grains. The basic structure and dynamic to economic life that began with grain agriculture has endured for 10 000 years and the duality between humans and Earth established therein is now reaching an apogee with the spectre of climate change and the mass extinction of other species on Earth. In this light, the questions emerge: Is the agricultural revolution an evolutionary transition adequately captured in existing frameworks of human social evolution? Is the human capacity for culture sufficient to override the power and dynamic of the economic superorganism?

This article is part of the theme issue ‘Human socio-cultural evolution in light of evolutionary transitions’.

## 1. Introduction

Evolutionary biology recognizes the importance of culture in explaining human social evolution. Group- and multi-level selection, gene–culture coevolution and now cultural multi-level selection help to frame the discourse on human social evolution and the evolution of cooperation [1–3]^1^. In this framework cultural variation captures the differences in human societies that we encounter, from hunter–gatherers to modern global capitalism, but it is the evolution of the capacity for culture that denotes the major evolutionary transition for humans. This essay offers observations from an economist that might lead evolutionary biologists to expand the complex landscape of human social evolution beyond the realm of culture. The example of agriculture (grain agriculture in humans and fungi agriculture in many species of ants and termites) is highlighted in order to place this economic system change as something distinct in the evolution of cooperation, where individuality *vis à vis* the group is fundamentally altered and a new whole emerges. The human transition to grain agriculture was clearly a major economic transition but is it sufficient to view it as a variant of culture? Should it rank among evolutionary transitions [4]^2^?

E. O. Wilson considers both humans and some insects as uniquely social species that have been extremely successful; they have literally taken over the world [5]. In advanced insect superorganisms, an extended division of labour emerges, often with morphological differentiation among members of the colony, and there are sterile workers that have clearly sacrificed their individual reproductive fitness for the benefit of the colony. The superorganism is a form of extreme sociality and cooperation. This is specifically what Hölldobler & Wilson mean when they refer to ‘close cooperation’ in insect species. In their words: ‘The basic elements of the superorganism are not cells and tissues but closely cooperating animals' [6, p. 4]. The superorganism denotes a unique level of hierarchy in the formation of life. There are clearly advantages to this level of organization, as measured by the success of these insect populations.

This type of extreme sociality does not occur in humans, so humans do not attain the status of a superorganism. Instead, the presence of advanced sociality in humans takes form through the evolution of the capacity for cooperation and, by extension, culture. Culture makes possible modern complex societies where technological advancement is cumulative and extensive cooperation occurs among people who are not related. Peter Richerson & Robert Boyd have helped to build the framework known as gene–culture coevolution [2]. The idea is that humans evolved a social psychology that enabled culture and its transmission through social learning. Kevin Laland elaborates this when he tells us that human beings possess ‘high-fidelity transmission mechanisms including an unusually accurate capacity for imitation, teaching and language’ [7, p. 174]. These facilitate the evolution of the capacity for culture and its cumulative aspect [7]. Humans have a unique ability for building and expanding on previous knowledge, adding to the cumulative cultural endowment.

The social hardware necessary for the development of cooperation and, by extension, culture is genetically based, evolving out of group- and multi-level selection and a dialectical interplay between the evolving capacity for culture and genetic change [1,5]^3^. Herbert Gintis states this well: ‘We are the species that we are because … genes provide individuals with the capacities and incentives to transform culture, and culture guides the transformation of the gene pool from generation to generation’ [8, p. 2]. Culture then provides humans with an adaptive flexibility. The capacity for culture (its replication and modification) is advantageous because it acts much like genetic change, only quicker. This was especially significant during the climactic changes occurring during the Pleistocene. The process is ongoing. Joseph Henrich tells us ‘Recognizing that we are a cultural species means that, even in the short run (when genes don't have enough time to change), institutions, technologies and languages are coevolving with psychological biases, cognitive abilities …. In the longer run, genes are evolving to adapt to these culturally constructed worlds … ’ [3, pp. 314–315]. Henrich goes on to posit the development of a collective brain ‘that depend[s] heavily on the packages of social norms and institutions that weave together communities, create interdependence, foster solidarity and subdivide our cultural information and labor’ [3, p. 318].

No-one doubts that the human expression of cooperation and sociality is unique to humans by virtue of the evolved human capacity for culture making us more cooperative, just as the expression of cooperation is unique in insects that gain the status of superorganism. The evolution of the capacity for culture was an evolutionary transition just as the evolution of the superorganism was an evolutionary transition in insects. In both cases, the advanced sociality was achieved through genetic changes that gave rise to cultural humans and insect superorganisms. Once humans attained culture, the pressure on genetic change is less significant and adaptation can take place through the flexibility afforded through cultural change. It is also the case that over time it is culture that influences genetic change, so there is dialectical process at play [9].

Unfortunately, the discussion of the evolution of human sociality and cooperation appears to have come to a halt there and the influence of gene–culture coevolution has become the catchall for vast differences in the expression of human sociality and cooperation. This narrative imbues humans with what might be an exaggerated sense of their power to cooperate and build on knowledge in order to bring about necessary change. There is no place where this cultural hubris is more evident than with the discourse on our present war between economy and Earth. The global economic system at play is bringing about the sixth mass extinction and unmitigated climate change and we continue to tinker around the edges of altering its structure and dynamic in any significant way. One could easily make the claim that it is the global economic system that has the upper hand and not our capacity for cultural change.

## 2. The agricultural revolution

It was the practice of agriculture by many species that forced me to think more expansively about the evolution of cooperation, evolutionary transitions, and the place of economic systems in the complex web of evolution. Here I am referring specifically to the cultivation of annual grains in humans (and fungi in insects). There were two things that captured my curiosity about agriculture. First was simply the dramatic change that agriculture was for the collective life of *Homo sapiens*. It changed the entire structure and dynamic of human material life, altering the relationship among humans and between humans and the more-than-human world, and it fundamentally altered the energetics of human society. It was a new whole in cooperative life. As an economist, it is apparent that the transition to agriculture was a major economic revolution. It is also clear that humans remain on the material trajectory set by the agricultural revolution (expansionary, materially interdependent with an elaborate division of labour, and forming a structural duality between humans and Earth). Global capitalism can be viewed as an elaboration of the economic system change that began with grain agriculture. Second, humans are not the only species to have made the transition to agriculture. All species that made this transition exhibit the same structure and dynamic as agricultural life (material organization). In this sense agriculture is a powerful and universal system and once it is engaged it carries the species along, so to speak.

It is illuminating to juxtapose the human agricultural system with what came before in order to appreciate the fundamental change in social/material organization brought about by agriculture.

Here I paint a broad stroke and my purpose is to help decipher the basic building blocks of an agricultural system [10]^4^. Until the agricultural revolution, humans had been a Pleistocene species and they were organized in a hunting and gathering economic system. Hunting and gathering is an economic system, that is to say, a system of material reproduction, where individuals are materially self-reliant (each individual had the knowledge and skill to fend for themselves), observant, minimalist and non-expansionary [11]^5^. Material survival was based on teaching and learning and a broad knowledge base, all embodied in the system of gathering and hunting. In this economic system, *H. sapiens* take their place as one of many species on Earth and to a great extent their material life is dictated by the rhythm and dynamic of the more-than-human world. In this way, they must be considered an embedded species. The feedback loops dictated by the imperative of mobility kept population density and growth rates low and limited material expansion. In fact, by the time humans began the practice of cultivation of annual grains the total human population on Earth stood at around 6–10 million people. One might say that hunting and gathering is an energetically contained system and not an energetically expansionary system. *Homo sapiens* resided in this type of economy for tens of thousands of years before they were collectively altered by the agricultural revolution beginning 10 000–12 000 years ago. Once humans began the practice of grain agriculture, the trajectory, dynamic and organization of their economic life fundamentally shifted. This was not a continuum along a path, rather it was a dramatic break from the past.

Annual grain agriculture stands in contradistinction to hunting and gathering. Grain agriculture is a system that has distinct feedback loops between cultivation, division of labour, and population that are mutually reinforcing and positive, making humans materially and therefore existentially interdependent and expansionary around the focal point of grain cultivation. In this way a duality developed between the agricultural system and the human relationship to Earth, limiting the fluid interchange with the rhythm and dynamic of the more-than-human world to annual cycles around the production of annual grains. James C. Scott tell us that humans were ‘disciplined and subordinated to the metronome of our own crops …. Once Homo sapiens took that fateful step into agriculture, our species entered an austere monastery whose task master was mostly the genetic clockwork of a few plants' [12, p. 91].

Thus, grain agriculture changed the expression of human cooperation, the collective energetics of the human species, and the human relationship with the more-than-human world and each other. It formed an economic system that had an insular and self-referential integrity to it that might be considered a unique whole in the evolution of human cooperation. In some sense this is the system that is with us now, although that may not seem obvious. Global capitalism can be considered ‘a system within a system’ in the sense that it models the agricultural system in its structure and dynamic—it is expansionary, characterized by profound material interdependence, and it is a system that brings the duality between humans and the more-than-human world to its apogee. The capitalist system is at once removed from the Earth, functioning in a self-referential manner (think of the circular flow of income and spending), and at the same time a profoundly material system [10]^6^. Clearly this is a dangerous variant of the agricultural system on an Earth that is limited.

I would have been inclined to leave the agricultural revolution to the framework of culture had it not been for one simple fact: humans are not the only species to practise agriculture. The cross-species exploration of agriculture took me to social insects, specifically many species of ants and termites that practise agriculture [13–16]^7^. Insects cultivators develop an aptitude for collective material life around the cultivation of fungi. In *H. sapiens* that aptitude is expressed around the cultivation of annual grains. Many insect species achieved the status of superorganism, and humans achieved the capacity for culture before the advent of agriculture, so a pre-existing level of evolved sociality was a precondition for an agricultural transition. In all these species a sociality that enabled the deployment of a division of labour does not require agriculture, but the division of labour becomes more extensive with agriculture, leading Hölldobler & Wilson to refer to the insects that practise agriculture as an example of an ‘extreme insect superorganism’ [17]^8^. The questions posed here are where to place the transition to agriculture in the evolutionary history of humans; where to place agriculture, a particular type of economic system, in the complexity of evolution; and finally whether it is correct to subsume the agricultural transition in humans into the evolution of culture.

The fact that agriculture is not the exclusive domain of humans is curious, but the more important point is that the structure and dynamic of material life around agriculture are unique, and the same for all species that made the transition to agriculture. All species involved in agriculture engage a coevolution that plays on and enhances the inherent tendencies of the species that cultivate—this is especially apparent in the expansion of their capacity for a division of labour but also in their coevolution with the species they cultivate. All engage positive feedback loops that are expansive. That process can be understood for humans in this way [6]. Early agriculture required an expansion of the division of labour simply because it was attached to a pre-existing system of hunting and gathering, so the number of tasks in day-to-day life was additive. As well, grain production is inherently expansionary owing to the fact that grains can be stored and the production in any given year is uncertain. Production in any given year is pushed to a maximum to guard against this year-to-year uncertainty. Surplus production is a foundation of grain agriculture and surplus further expands the division of labour in humans. In simplistic terms it opens the door for many occupations that are not directly engaged in agricultural production. It is also the case that grain production is standardized through the coevolution of all species involved. This too expands and regiments the division of labour. For example, the selection of non-shattering seeds may have happened without any intent but it served to standardize grains and their production. Standardization provides a way to capitalize on the efficiency inherent to a division of labour. The production of annual grains becomes a more integrated and regimented process [10]^9^. The division of labour coalesces around the focal point of cultivation and by extension its surplus, thereby forming a powerful feedback dynamic between it, cultivation and population growth. This is the basic (and universal) architecture of the agricultural system that gives the system integrity and assures its reproduction [6]^10^. Agricultural species become a materially integrated whole and individual autonomy in material life is diminished, if not eliminated altogether. Individual survival is centred on engaging in the collective agricultural enterprise.

The exploration of the unique expression of sociality and cooperation found in human agriculture has not been expansively entertained by evolutionary biologists except insofar as it is subsumed under the evolution of culture, the important evolutionary transition to “Generally evolutionary biologists think of agriculture in humans as a cultural transition.”^11^ David Sloan Wilson says ‘the advent of agriculture enabled us to increase the scale of society by many orders of magnitude through a process of cultural multilevel selection’ [9, p. 90]. But as a matter of significance in light of evolutionary transitions, agriculture has not been considered noteworthy. Rather, it is a sidebar to the history of the social evolution of humans—an evolution that is demarcated by the evolution of the capacity for culture. The exception to this is Joseph Henrich, who sees that culture (and gene–culture coevolution) has led to an extension of the division of labour and interdependence, moving humans in the direction of a ‘superorganism of a sort’ [3, p. 318]. It seems he would consider this trend part of a major evolutionary transition, but he does not connect this directly to agriculture—for him there is a continuum of this interplay over time.

The facts stand before us—the structure and dynamic of cooperation expressed in an agricultural system are unique, universal, powerful, and engaged by species that do not have what we would consider culture. The agricultural system fundamentally alters the relationship of the individual to the group and through its powerful feedback loops and cohesion it assures its own reproduction. Those who try to explain the agricultural revolution as a subset of species-specific evolutionary histories eliminate the ability to register the significance of agriculture as a powerful and universal system and to place that significance in the complex web of evolution. Again, let me reiterate the importance of this for humans. Humans are now organized in a global economic system that is difficult to alter in meaningful ways—if by meaningful we mean in ways that halt the increase in global temperature and the sixth mass extinction. The basic architecture of the agricultural system clearly has tremendous staying power despite what we might consider many cultural variants and the presence of human prosociality.

## 3. The economic superorganism

Agricultural groups become extremely successful in expanding their numbers, and all form a highly integrated productive whole. For want of a better term we can label all the species that engage agriculture *economic superorganisms* [10]^12^. Again, I am making the distinction here between superorganisms as described and defined by E. O. Wilson, Hölldobler and others and used loosely by Henrich, and an economic superorganism, which refers specifically to the structure and dynamic in cooperative material life particular to agriculture. The term 'economic superorganism' is not to be interpreted as biological (although in insects there is a biological component in the sense that the formation occurs through mutation and selection in insects over a long span of time). It refers to the cohesive whole brought about by agriculture and the architecture that underlies it. Some insect species are superorganisms, although all insect superorganisms do not practise agriculture. No humans are superorganisms in the way insects are, but some insect species and most of humanity became economic superorganisms when they engaged agriculture.

Economic superorganisms are joined in a vast enterprise of cultivation affecting the fitness of the group, the integrity of the individual *vis à vis* the group, and the relationship of the group to the world outside the group. It is a powerful and enduring system. There is no category for economic superorganism in the lexicon of evolutionary biology nor any particular evolutionary distinction to this transition. This owes partly to the preoccupations of those that study sociality and extreme cooperation. The mechanisms of engagement (culture and genetic change through mutation and selection) of the agricultural system hold centre stage in discussions of the evolution of cooperation and sociality, but in the context of the transition to agriculture they might more accurately be considered players in something more significant.

Genetic changes we observe in the formation of insect agriculturalists and culture in humans might be accommodating to (and masking) the force and importance of the ascendant agricultural system. Pre-existing sociality and capacity for cooperation facilitate the engagement of this new variation on the energetic and cooperative life of the species that practise it. It seems that if we are to seriously explore the emergence of agriculture across species and the similarities we find therein, the aperture of evolution will have to be expanded to consider that the system of agriculture is its own force, an emergent and distinct cooperative whole, and something that might have standing as an evolutionary transition.

So distinct and powerful is this system formation that genetic change and culture might be considered mechanisms of engagement hijacked by the emergent economic superorganism. I realize I am proposing something that is outside the present range of discussions around social evolution of humans. Historically the agricultural transition has been subsumed under a pre-existing framework of social evolution—broadly in the framework of the evolution of the capacity for culture and gene–culture coevolution. An extensive division of labour around the focal point of cultivation and the attendant feedback loops are the most salient characteristics of the architecture of cooperation in an agricultural system. This is not particularly complicated. Mutation and selection (a genetic matter) as found in insect agriculturalists may help to facilitate the extension of labour around the agricultural system, but in a broader sense it is simply one mechanism for engaging the extreme role differentiation that accompanies all agricultural species. In humans, the division of labour is enabled through the capacity for culture and our uber sociality (that we are programmed to be cooperative), so the nascent and expansive division of labour that accommodates agriculture occurs more quickly. In other words, multiple mechanisms can be used to facilitate the formation of an extensive division of labour that coalesces around the focal point of cultivation. The division of labour then becomes a crucial stanchion and one of the major feedback loops in the structuring of the agricultural system.

Through the emergence of this particular and unique system the evolutionary histories of diverse species are pulled in the same direction. In other words, it is the emergence of an economic structure and dynamic particular to the agricultural system that bears down on the evolutionary path of species with a certain evolved disposition toward sociality. To say then that agriculture is merely a specific expression of an insect superorganism or a specific extension of the human capacity for culture does not give the agricultural system its due. The human capacity for culture which includes the deployment of the division of labour works quickly to engage the agriculture system but the agricultural system is not merely an artefact of or extension of the human capacity for culture. In fact, it may influence the direction of cultural evolution in a way that assures that all aspects of culture support the foundational structure and dynamic of the agricultural system.

A few words about the division of labour and its role in the choreography of the agricultural system are in order. Economists have long understood that there are efficiencies to the division of labour that are universal and undeniable. Biologists studying insect superorganisms note the same. Efficiencies may influence the selection process toward a more elaborate division of labour for both insect superorganisms that practise agriculture and human societies that engage agriculture. The focus on efficiency, however, should not eclipse the more important fact that the division of labour around the focal point of grain cultivation (or fungi cultivation) provides cohesion to the nascent agricultural group. This cohesion provides integrity to the agricultural system and it is an integral part of its power and formation as a distinct whole. The system becomes more structurally interdependent as a result of the division of labour around cultivation. The evolutionary biologist Peter Corning refers to the division of labour as the paradox of dependency. He tells us: ‘… there is a deep paradox involved in dividing up and sharing the elements of a job. It creates an interdependency; everyone must do their part or the desired outcome will not be achieved.’ The division of labour creates ‘a built-in enforcer for cooperation’ [18, p. 50].

Clearly the deployment of a division of labour and role partitioning and coordination need not rely on unique attributes of human culture because insect economic superorganisms do it without culture, and by noting this fact we shift our focus from the mechanisms of engagement to the pull and power of the new whole in the form of the agricultural system [19].^13^ I might note as an aside that insects often communicate through chemical signals so they do not have culture but they have evolved the capacity for communication, which facilitates the deployment of certain aspects of their division of labour. One way to look at the division of labour more expansively is that, like social learning, the division of labour is a potentiality that crosses species boundaries. Just as human culture is a unique expression of the universal tendency toward social learning, agriculture is a unique expression of the universal tendency toward a division of labour. The agricultural system is a particular play on the sociality of species where that sociality and propensity for cooperation is restructured into an economic superorganism, a very particular and powerful economic system.

The transition to agriculture is a significant change in the history of life on Earth and certainly in the history of human evolution. For humans this alteration in their material ordering created a duality between the human economic system (the economic superorganism) and the Earth. Let me reiterate, global capitalism is the legacy of the agricultural system. It is an elaboration of the agricultural system. Surplus and expansion and profound, almost mechanistic, interdependency in material life, and duality in the human relationship to the more-than-human world became the order of the day beginning with grain agriculture. The basic structure and dynamic of the agricultural system were subsequently extended with elaborations that have eventually led to global capitalism. This type of elaboration does not occur in insect agriculturalists and in this way their feedback loops are more contained and less complicated.

To make this more understandable, think of the evolution of markets not as an outcome of any natural propensity to truck, barter and exchange embedded in human nature, as Adam Smith would have it, but as the result of surplus generated in an agricultural system. Markets are an institutional elaboration of surplus created in an agricultural system but they also expand and elaborate the economic system. Markets change over time from a place where surplus is traded to a market economy where surplus in the form of surplus value is generated in production and realized in sale where it becomes profit. Generating profit and reinvesting become imperatives of the system, a requirement for staying in the game. This should be understood as something more foundational than simple greed. Capitalism functions according to its own internal logic, a logic that is disconnected from the ecologies of the Earth even as it is a fundamentally material system. To understand this, simply think about all the economic variables that are interrelated and react with one another. An economy can become depressed not because it runs out of resources but by a problematic linkage between savings and investment for example—this is part of the internal logic of the system.^14^ Global capitalism fertilized with the use of fossil fuel brings the duality between economy and Earth that began with the agricultural system to its apogee. Constraints to expansion imposed by energy are temporarily removed and the internal logic of the system is left to play itself out independent of biophysical constraints [10]^15^. We are just now experiencing that problematic duality (and the necessity of reconnection) with climate change and the sixth mass extinction.

We might ask whether the unique capacity for sociality and its grounding in culture in humans was hijacked through the engagement of an economic superorganism that in the end works against the continued evolution of human intelligence and violates the best impulses of human cooperation—that is, to live in ecological community. This runs contrary to the belief that humans are on a continuum of gene–culture coevolution that is bringing about a new major transition into a ‘new kind of animal’ where we are becoming increasingly prosocial. That is to say, humans are becoming ‘more attentive parents, loyal mates, good friends (reciprocators, and upstanding community members' and ‘possess institutions that make group level decisions’ [3, p. 318]. This is worth pondering, particularly given the force of the global economic system. The force of this system in its present form is so formidable that we cannot seem to manage it or disengage from it despite our intelligence, and institutional and cultural capacities. Humans play on the universal with their particular cultural capacity but the system should not be understood as a mere expression of culture. The danger of viewing the system solely through an aperture of culture and increasingly prosocial behaviour is that it distorts our perception of our power to alter it and it distorts our perception of the significance and tenor of this change in collective material life. Though the agricultural revolution and its evolution in the ensuing 10 000 years has produced an insignificant change in the genetic endowment of humans, it is producing a profound change in the aggregate genetic endowment of life on Earth. This is a ‘momentous consequence’ of the agricultural revolution.^16^ *Homo sapiens* have become engaged by a collective strategy for survival that has been (at least temporarily) extremely successful in the measurement of their fitness and extensive interdependence while possibly assuring long-term collapse, but then evolution does not see ahead.

## 4. Conclusion and implications

More refinement if not deconstruction of cooperation and social evolution in light of the agricultural revolution and the economic system put in play with this revolution is in order. A deeper understanding of the place of this particular system change in the complexity of evolutionary transitions is a challenge for evolutionary biology. Culture and its power in prosocial evolution are the continuing preoccupation of those who are interested in human social evolution. But we might ask whether that preoccupation has discouraged a more expansive understanding of economic system dynamics and their role in social evolution. Is the engagement of an agricultural system a new whole in collective material life—and if so where should it be positioned in the landscape of evolution? Is it merely the result of gene–culture coevolution or something more? Have we become *H. sapiens agriculturii*, all members of the economic superorganism [10]^17^?

These are important questions for evolutionary biology and transdisciplinary studies surrounding human cooperation, but there is a more practical question that emerges from this focus. Given the power of the agricultural system and its legacy in global capitalism, can humans intentionally undo the whole that was created with the engagement of grain agriculture and amplified over the past 10 000 years? This question bears down on the tension surrounding our prosocial evolution. The altered strategy of material life begun with agriculture has had such force that it has guided the formation of institutions, ideologies, and innovation practised by humans in the ensuing 10 000 years, and not the other way around. Culture, and likely any genetic change it manages, serves to enhance the economic superorganism and not to effectively counter it, and yet it appears that it does need to be altered in foundational ways.

Material systems (economic systems) of species are the product of dialectics of evolution and the complexity of emergent systems played out on Earth. Of course, culture and its interplay with genes are involved for humans, but so is the complexity of systems organization that gives rise to something distinct and powerful: an economic system. The universality of the agricultural system and its particular and universal play on sociality is unsettling. Sociality moves in a certain direction around cultivation and becomes an integral and structural part of the agricultural system (the economic superorganism), and we begin to look very similar to agricultural insects. This is not exactly what those touting the increasingly prosocial evolution of humans had in mind. Again, theirs is mostly a positive spin on the ‘new kind of animal’ being created through the ongoing processes of human evolution [3]. The focus presented here reorients this discussion as well as that of determinism versus potentiality [20]. An economic system might be more deterministic of human behaviour than we have been inclined to believe despite the flexibility in social expression that humans are imbued with by virtue of their capacity for culture. And the prosocial evolution of humans may be more mechanistic and deterministic in a way that runs counter to the positive spin customarily given to our social evolution.

The agricultural system, the economic superorganism, moved *H. sapiens* away from being an observant mammal embedded in the rhythm and dynamic of the Earth in the execution of daily life to a collective life more reminiscent of social insects that practise agriculture. Expansion and interdependence that reduces if not eliminates individual autonomy crosses species boundaries, and in *H. sapiens* surplus, hierarchy and a profound and structural duality between the economic superorganism and Earth make the collective life of humans its own distinctive whole. Is this a major evolutionary transition for humans that has fallen under the radar of the discourse in evolution? It is a good exercise for humans to humble themselves sufficiently to acknowledge that their social evolution is not that different from that of agricultural insects. This is not meant to engender hopelessness. It is meant to engender reflection on the difficulty of change in the shadow of such a formidable system. In this space we might begin to question how much power we have to alter things, and when we begin to ask that question we might be more inclined to stand back and think more seriously about how to regroup. Here we come to understand that this foundational change puts us face to face with our complex evolutionary history but it is a different evolutionary history from that we had in mind. Here we might pose a tautological question: Can our social evolution save us from the path of our social evolution that began 10 000 years ago?

## Data Availability

This article has no additional data.

## Endnotes

1 See for example [1–3].

2 For an overview of evolutionary transitions see [4].

3 Some of the most heated debates in evolutionary biology have emerged over the evolution of sociality. Group and multilevel selection have taken their place as guideposts in understanding the evolution of extreme cooperation, so much so that E. O. Wilson claimed: ‘The old paradigm of social evolution, grown venerable after four decades, has thus failed …. kin selection, if it occurs at all in animals, must be a weak form of selection that occurs only in special conditions easily violated’ [5, p. 181]. E. O. Wilson & David Sloan Wilson outline the landscape of group selection as it now stands: ‘… for the social group to function as an adaptive unit its members must do things for each other. Yet, these group-advantageous behaviors seldom maximize relative fitness within the social group. The solution according to Darwin is that natural selection takes place at more than one level of the biological hierarchy’ [1]. My purpose here is not to resolve the tension between group and kin selection.

4 I follow the discussion laid out in my book: [10].

5 This does not mean that humans did not work collaboratively or that it was not of benefit (a selective advantage) to do so. Michael Tomasello and coauthors discuss their interdependence hypothesis, where they argue that humans were obligate collaborative foragers and this helped to select for their particular aptitudes as cooperators. See [11].

6 For an extensive exploration of capitalism as a ‘system within a system’ see [10].

7 John Gowdy and I explored this similarity extensively. See [13–16].

8 Hölldobler & Wilson [17]. It should be noted that before agriculture the division of labour in humans was based primarily on age and sex.

9 For a more extensive discussion of the expansion of the division of labour with agriculture, see [10].

10 The elaboration of the division of labour in insect agriculturalist is discussed in detail by Hölldobler & Wilson [6].

11 Hölldobler & Wilson [6] are very clear that insect agriculturalists develop the most complex of the societies of the biological superorganisms so they do acknowledge agriculture in this sense. The leafcutter ants are the best example of the elaboration of the superorganism around agriculture.

12 *Homo sapiens agriculturii* is terminology introduced in Krall [10].

13 Gordon [19] has an extensive and critical discussion of the division of labour. She claims the terminology is misleading and the formation of collective behaviour needs to be more expansively studied.

14 The understanding of the internal logic of capitalism depends on the school of economic thought to which one adheres.

15 For a more detailed discussion of the internal logic of capitalism and the duality it presents between economy and Earth, see Krall [10].

16 David Sloan Wilson identifies a ‘momentous consequence’ as one of the attributes of a major evolutionary transition.

17 This is terminology is introduced in [10].
